# Applying a multi-task and multi-instance framework to predict axillary lymph node metastases in breast cancer

**DOI:** 10.1038/s41698-025-00971-0

**Published:** 2025-06-18

**Authors:** Yizhi Li, Zonglin Chen, Ziyuan Ding, Danyang Mei, Zhenzhen Liu, Jia Wang, Kui Tang, Wenjun Yi, Yan Xu, Yixiong Liang, Yan Cheng

**Affiliations:** 1 Department of Pharmacy, The Second Xiangya Hospital, Central South University, Changsha, 410000 China; 2 Hunan Provincial Engineering Research Centre of Translational Medicine and Innovative Drug, Changsha, 410000 China; 3 Department of General Surgery, The Second Xiangya Hospital of Central South University, Changsha, 410000 China; 4 Clinical Research Center for Breast Disease in Hunan Province, Changsha, 410011 China; 5 School of Computer Science, Central South University, Changsha, 410006 China; 6 The Affiliated Cancer Hospital of Zhengzhou University & Henan Cancer Hospital, Zhengzhou, 450008 China; 7 Department of Ultrasound Diagnosis, The Second Xiangya Hospital of Central South University, Changsha, 410000 China

**Keywords:** Cancer, Computational biology and bioinformatics, Medical research, Oncology, Risk factors

## Abstract

Deep learning (DL) models have shown promise in predicting axillary lymph node (ALN) status. However, most existing DL models were classification-only models and did not consider the practical application scenarios of multi-view joint prediction. Here, we propose a Multi-Task Learning (MTL) and Multi-Instance Learning (MIL) framework that simulates the real-world clinical diagnostic scenario for ALN status prediction in breast cancer. Ultrasound images of the primary tumor and ALN (if available) regions were collected, each annotated with a segmentation label. The model was trained on a training cohort and tested on both internal and external test cohorts. The proposed two-stage DL framework using one of the Transformer models, Segformer, as the network backbone, exhibits the top-performing model. It achieved an AUC of 0.832, a sensitivity of 0.815, and a specificity of 0.854 in the internal test cohort. In the external cohort, this model attained an AUC of 0.918, a sensitivity of 0.851 and a specificity of 0.957. The Class Activation Mapping method demonstrated that the DL model correctly identified the characteristic areas of metastasis within the primary tumor and ALN regions. This framework may serve as an effective second reader to assist clinicians in ALN status assessment.

## Introduction

Breast cancer is the most commonly diagnosed malignancy among females worldwide^1^. A majority of breast cancers are detected at an early stage, making surgery a cornerstone for their management. Axillary lymph node (ALN) status is a critical factor influencing surgical planning and determining the need for neoadjuvant treatment. The presence of ALN metastasis is also the most important predictor of overall recurrence and survival for breast cancer patients^2,3^. Therefore, a precise determination of ALN status is imperative for effective breast cancer management. In routine clinical practice, the detection of ALN status is typically through presurgical ALN biopsy for those with palpable ALNs or surgical ALN dissection for those with clinically negative ALN. However, both of the procedures are invasive, and in some cases, the axillary surgery is unnecessary for patients who were sentinel lymph node positive but ALN negative^4^. Hence, there has been active exploration on developing noninvasive approaches capable of preoperatively discerning ALN metastasis, hoping for improving clinical axillary management.

Ultrasound examination is a preferred method for breast cancer preoperative assessment since it can provide a direct visualization of both the primary tumors and ALNs in a convenient, cost-effective and harmless manner^5,6^. Several ultrasound ALN manifestations, such as enlarged lymph nodes, irregular shapes, hypoechoic appearance, and loss of fatty hilum, are suspicious signs indicative of metastasis. Additionally, certain ultrasound features of breast cancer, including tumor size and distance of breast cancer from the skin and the nipple, can also suggest ALN metastasis. However, solely relying on these characteristics to diagnose ALN status can often lead to inconsistence and misdiagnosis, as it heavily depends on the experience of individual physicians, and naked eye inspection alone sometimes overlooks image details indicative of micrometastasis^7^. In this regard, algorithmic models such as clinical-pathological nomograms and radiomics-based models have been developed to predict ALN status. However, these models have shortcomings in clinical application. Nomogram models often achieve high diagnostic efficacy only after incorporating postoperative pathological parameters into the model^8^. Radiomics, which relies on machine learning algorithms for the model construction, can quantify ultrasound manifestations from different perspectives; however, the quantification process requires time-consuming manual pixel-by-pixel delineation, which also makes this method difficult for clinical translation^9,10^.

Artificial intelligence, particularly deep learning (DL) models, is expected to change the diagnosis and treatment landscape due to their abilities to recognize even subtle features that hold predictive significance on medical images and make quantitative evaluations in a reproducible and labor-free way^11–13^. It has proven effective in various medical applications. For instance, Jiang et al. successfully constructed an effective DL model using CT images as input to predict prognosis and cancer immunotherapy response^14^; Qian et al. developed a DL model for prospective assessment of breast cancer risk from ultrasound images, with the AUC exceeding 0.9^15^. Specifically, using ultrasound images for DL model construction also proved to achieve satisfactory outcome for predicting breast cancer ALN status in previous studies^16,17^. These studies have well demonstrated the feasibility of DL models for different clinical diagnostic purposes in breast cancer.

Despite these promising results on DL, its real-world utility is hindered by various factors. First, DL is often criticized as a black box that lacks interpretability. This makes clinicians confused about its outputting outcomes, and doubt whether the model had really learned the intrinsical image features. Second, clinical image diagnosis is often based on multi-view images. However, many of the existing DL models did not consider this practical application scenario^17–19^, which is easy to cause bias. Importantly, there are no DL models incorporating both the primary tumor and ALN ultrasound images for predicting ALN status. The main reason for this phenomenon is that ultrasound examination sometimes doesn’t detect a visible ALN for a part of clinically ALN negative patients. However, this is unreasonable since the ultrasound ALN manifestation is the main criterion for presurgical ALN status diagnosis, and relying solely the primary breast cancer images to predict ALN metastasis is unconvincing.

Hence, we aimed to propose a DL model to predict ALN status for breast cancer patients that simulates the real-world clinical diagnostic scenarios. We suppose this DL model to use both the primary and ALN images for ALN prediction for those with visible ALNs, and primary breast cancer images alone for those with ALN unseen to ultrasound examination. To achieve this goal, we designed a two-stage DL framework. At the first-stage, we used a multi-task learning (MTL) framework to simultaneously detect the primary tumor and ALN areas within the ultrasound images and make an image-level prediction on ALN status. By integrating lesion segmentation into the prediction process, our model is constrained to learn features from clinically significant areas, which can enhance clinical trust to some extent. In the second stage, to mimic the real-world scenario of ALN diagnosis, we adopted a multi-instance learning (MIL) framework to integrate the image features from different tumor and ALN (if available) lesions to make a patient-level diagnosis. Besides, for selecting the best performing model, different CNN and Transformer models were assessed respectively for image feature extraction to construct this two-stage DL framework.

## Results

### Clinical characteristics of the involved patients

The clinical characteristics of the patients involved in this study are depicted in Table 1. The median ages were 50, 49 and 51 years in the training, internal test and external test cohorts, respectively. The majority of the patients were diagnosed with clinical T1 or T2 stage and categorized into BI-RADS 4C and 5 tumor grades across the cohorts. Patient in the training and internal test cohorts had comparable characteristics regarding pathological indicators, including ER, PR and CerbB-2 status, Ki67 expression level, as well as lymphovascular and perineural invasions. However, more patients in the external cohort exhibited hormonal receptor positive, Ki67 level 51–100%, and lymphovascular invasion, suggesting population heterogeneity during the retrospective patient enrollment process in different clinical centers. The distribution of the clinical characteristics in breast cancer patients with or without ALN metastasis is listed in Table S1. Of the enrolled patients, 64.2% (*N* = 735), 67.4% (*N* = 184) and 67.1% (*N* = 94) were pathologically confirmed to have ALN metastasis in the training, internal test and external test cohorts, respectively. Compared to patients with negative ALN, these patients were more likely to have an advanced T stage and a higher ultrasound-reported BI-RADS grade in the training and internal test cohort, and were more likely to present with lymphovascular invasion in all three cohorts.

**Table 1:** Clinical characteristics of patients enrolled in this study

Characteristic	Training cohort (*N* = 1144)	Internal test cohort (*N* = 273)	External test cohort (*N* = 140)	*p* value^a^	*p* value^b^	*p* value^c^
Age, median (IQR)	50 (44, 57)	49 (44, 56)	51 (46, 57)	0.534	0.315	0.241
Clinical T stage						
T1	349 (30.5%)	92 (33.7%)	55 (39.3%)	0.196	0.028	0.346
T2	697 (60.9%)	164 (60.1%)	81 (57.9%)			
T3	66 (5.8%)	15 (5.5%)	4 (2.9%)			
T4	32 (2.8%)	2 (0.7%)	0 (0%)			
Ultrasound reported BI-RADS				**0.036**	**<0.001**	**0.030**
4A	66 (5.8%)	29 (10.6%)	19 (13.6%)			
4B	227 (19.8%)	51 (18.7%)	16 (11.4%)			
4C	438 (38.3%)	103 (37.7%)	70 (50.0%)			
5	413 (36.1%)	90 (33.0%)	35 (25.0%)			
Pathological type				0.080	0.067	**0.002**
Invasive ductal	924 (80.0%)	233 (85.3%)	102 (72.9%)			
Invasive lobular	22 (1.9%)	6 (2.2%)	5 (3.6%)			
Invasive cancer mixed with in situ cancer	171 (14.9%)	25 (9.2%)	31 (22.1%)			
Others	27 (2.4%)	9 (3.3%)	2 (1.4%)			
Receptor status						
ER status				0.763	**0.002**	**0.010**
Positive	739 (64.6%)	179 (65.6%)	109 (77.9%)			
Negative	405 (35.4%)	94 (34.4%)	31 (22.1%)			
PR status				0.297	**<0.001**	**<0.001**
Positive	601 (52.5%)	153 (56.0%)	102 (72.9%)			
Negative	543 (47.5%)	120 (44.0%)	38 (27.1%)			
CerbB-2 status				0.183	0.205	0.813
Positive	405 (35.4%)	85 (31.1%)	42 (30.0%)			
Negative	739 (64.6%)	188 (68.9%)	98 (70.0%)			
Ki67 expression				0.204	**0.014**	**0.004**
1–10%	102 (8.9%)	34 (12.5%)	5 (3.6%)			
11–50%	776 (67.8%)	178 (65.2%)	90 (64.3%)			
51–100%	266 (23.3%)	61 (22.3%)	45 (32.1%)			
Lymphovascular invasion				0.968	**<0.001**	**<0.001**
Yes	279 (24.4%)	65 (23.8%)	72 (51.4%)			
No	367 (32.1%)	87 (31.9%)	46 (32.9%)			
Unknown	498 (43.5%)	121 (44.3%)	22 (15.7%)			
Perineural invasion				0.918	0.504	0.512
Yes	85 (7.4%)	19 (7.0%)	9 (6.4%)			
No	537 (46.9%)	126 (46.2%)	73 (52.1%)			
Unknown	522 (45.6%)	128 (46.9%)	58 (41.4%)			

*IQR* interquartile range, *BI-RADS* breast imaging reporting and data system. Statistically significant P-values are highlighted in bold.

^a^Comparison between the training and internal cohorts.

^b^Comparison between the training and external cohorts.

^c^Comparison between the internal and external cohorts.

### Algorithm performance of the clinical model, DL frameworks using different CNN and Transformer models as network backbone

In the multi-task DL stage, four CNN models (HRNet, ResNet, Unet and MobileNet) and two Transformer models (Swin and Segformer) were respectively utilized as the backbone of the DL framework for image structure extraction. As shown in Table 2, the algorithmic performance of these models was assessed using indicators including the area under the curve (AUC), accuracy, sensitivity, specificity, positive predictive value (PPV), negative predictive value (NPV) and F1 score. Among the four CNN models, the HRNet and Unet models had higher AUCs, with 0.811 (95% CI: 0.751–0.872) for the HRNet model and 0.806 (95% CI: 0.752–0.860) for the Unet model, and both of these models were statistically superior to the clinical model (HRNet model vs. clinical model, *p* = 0.009; Unet model vs. clinical model, *p* = 0.007; DeLong test) in the internal test cohort. Similarly, both of the Transformer models achieved significantly higher AUCs than the clinical model, with 0.858 (95% CI: 0.812–0.904) for the Swin model and 0.832 (95% CI: 0.780–0.885) for the Segformer model (Swin model vs. clinical model, *p* < 0.001; Segformer model vs. clinical model, *p* < 0.001; DeLong test) in the internal test cohort. Besides, both the Transformer models achieved significantly higher AUCs than the single T model (Swin model vs. the single T model, *p* = 0.003; Segformer model vs. the single T model, *p* = 0.031; DeLong test). In the external test cohort, the CNN HRNet model still yielded a higher AUC than the clinical model (AUC: 0.823; 95% CI: 0.739–0.906; HRNet model vs. clinical model, *p* = 0.016; DeLong test), while the Unet model exhibited comparable performance with the clinical model. In addition, both the Transformer models outperformed the clinical model (Swin model vs. clinical model, *p* = 0.027; Segformer model vs. clinical model, *p* < 0.001; DeLong test), and the superiority was evident for the Segformer model, which exhibited a favorable AUC of 0.918 (95% CI: 0.869–0.967). It also had a significantly higher AUC compared to the single T model (*p* < 0.001).

**Table 2:** Algorithmic performance of the clinical model, the single T model and the two-stage frameworks using different CNN and Transformer models as network backbone

Methods	AUC (95% CI)	Accuracy	Sensitivity	Specificity	PPV	NPV	F1 Score	*p* value^a^	*p* value^b^
Internal test cohort									
Clinical model	0.681 (0.612–0.749)	0.678	0.685	0.663	0.808	0.504	0.741	Reference	0.263
Single T model	0.736 (0.667–0.805)	0.729	0.734	0.719	0.844	0.566	0.785	0.263	Reference
DL framework (CNN)^c^									
HRNet	0.811 (0.751–0.872)	0.824	0.864	0.742	0.874	0.725	0.869	0.009	0.010
ResNet	0.747 (0.687–0.808)	0.670	0.592	0.831	0.879	0.497	0.708	0.166	0.806
Unet	0.806 (0.752–0.860)	0.740	0.696	0.831	0.895	0.569	0.783	0.007	0.122
MobileNet	0.759 (0.699–0.820)	0.722	0.696	0.775	0.865	0.552	0.783	0.091	0.614
DL framework (Transformer)^d^									
Swin	0.858 (0.812–0.904)	0.813	0.810	0.820	0.903	0.676	0.854	<0.001	0.003
Segformer	0.832 (0.780–0.885)	0.828	0.815	0.854	0.920	0.691	0.864	<0.001	0.031
External test cohort									
Clinical model	0.652 (0.558–0.747)	0.664	0.723	0.543	0.764	0.490	0.742	Reference	0.159
Single T model	0.742 (0.657–0.827)	0.707	0.702	0.717	0.835	0.541	0.763	0.159	Reference
DL framework (CNN)^c^									
HRNet	0.823 (0.739–0.906)	0.807	0.819	0.783	0.885	0.679	0.851	0.016	0.228
ResNet	0.718 (0.612–0.823)	0.793	0.872	0.630	0.828	0.707	0.849	0.388	0.686
Unet	0.603 (0.506–0.699)	0.593	0.564	0.652	0.768	0.423	0.650	0.459	0.006
MobileNet	0.804 (0.726–0.883)	0.814	0.787	0.870	0.925	0.667	0.850	0.019	0.313
DL framework (Transformer)^d^									
Swin	0.801 (0.707–0.895)	0.850	0.904	0.739	0.876	0.791	0.899	0.027	0.382
Segformer	0.918 (0.869–0.967)	0.886	0.851	0.957	0.976	0.759	0.909	<0.001	<0.001

^a^Comparisons between clinical model and other models using DeLong test.

^b^Comparisons between single T input model and other models using DeLong test.

^c^Different CNN models as network backbone.

^d^Different Transformer models as network backbone.

**Fig. 1:**
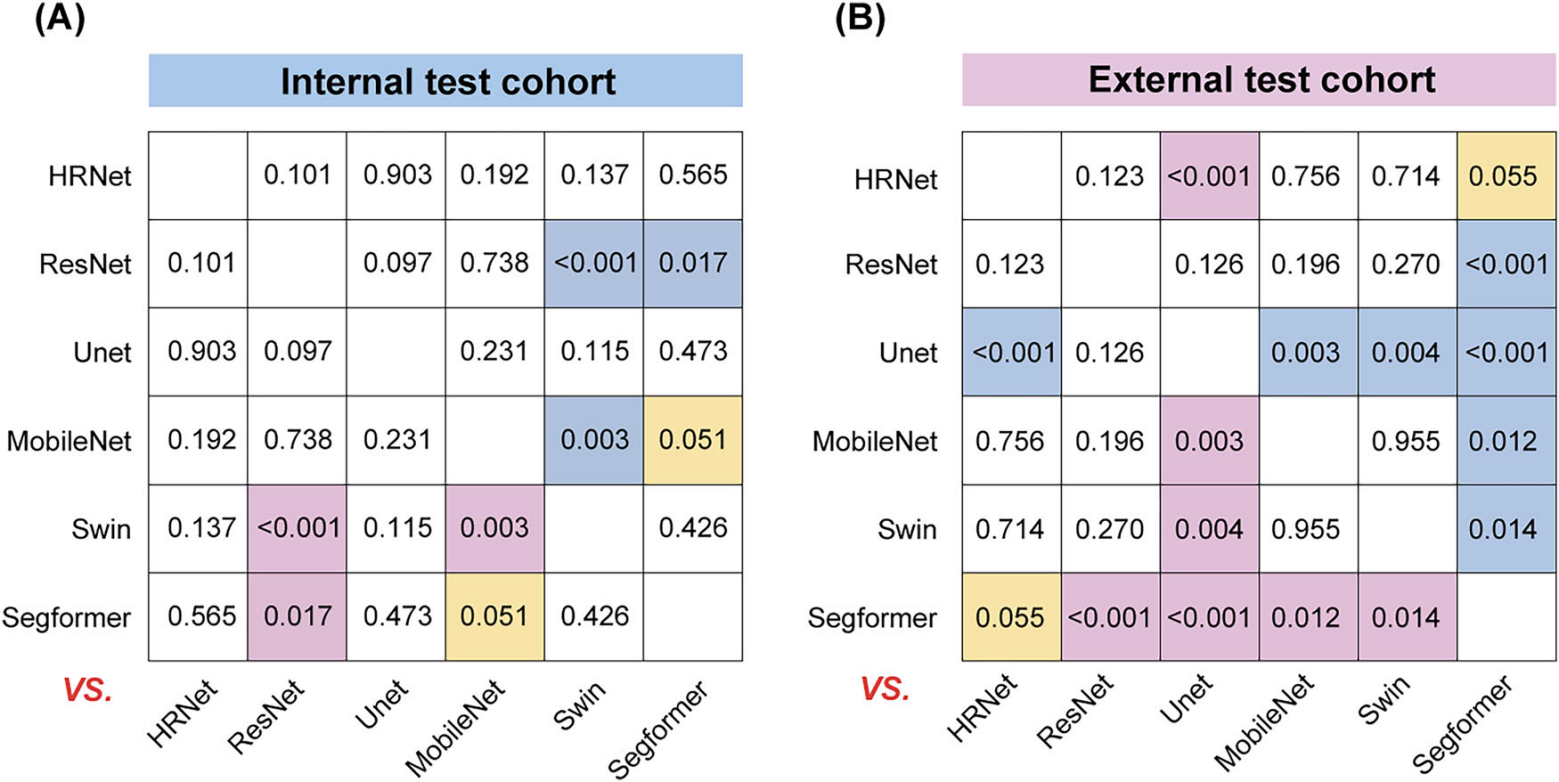
The DeLong test was used to compare the AUCs among the multi-task and multi-instance frameworks, using different CNN and Transformer models, respectively, as network backbone for image feature extraction. **A** AUC comparisons in the internal test cohort. **B** AUC comparisons in the external test cohort. Detailed DeLong *p* values are provided in each grid. Pink and blue colors respectively indicate significantly higher and lower AUCs, respectively, for the models annotated on the right side of the panel compared to those below. Yellow indicates a borderline *p* value. AUC area under curve, CNN convolutional neural network.

For the internal test cohort, among the six DL frameworks, accuracies ranged from 0.670 (the ResNet model) to 0.828 (the Segformer model); sensitivities ranged from 0.592 (the ResNet model) to 0.864 (the HRNet model); specificities ranged from 0.742 (the HRNet model) to 0.854 (the Segformer model); PPVs ranged from 0.865 (the MobileNet model) to 0.920 (the Segformer model); and NPVs ranged from 0.497 (the ResNet model) to 0.725 (the HRNet model). For the external test cohort, accuracies ranged from 0.593 (the Unet model) to 0.886 (the Segformer model); sensitivities ranged from 0.564 (the Unet model) to 0.904 (the Swin model); specificities ranged from 0.630 (the ResNet model) to 0.957 (the Segformer model); PPVs ranged from 0.768 (the Unet model) to 0.976 (the Segformer model); and NPVs ranged from 0.423 (the Unet model) to 0.791 (the Swin model). These indicators suggested that, numerically, the HRNet and Segformer models had relatively good performance than other CNN and Transformer models. Figure 1 summarizes the DeLong test *p* values of the comparisons between distinct MTL and MIL frameworks. Considering the superior discrimination abilities of the HRNet and Segformer models, we next used these two models as representatives of the CNN and Transformer models, respectively, for further analyses.

### Clinical performance assessments using receiver operating characteristic (ROC), precision-recall (PR) and decision curve analysis (DCA)

**Fig. 2:**
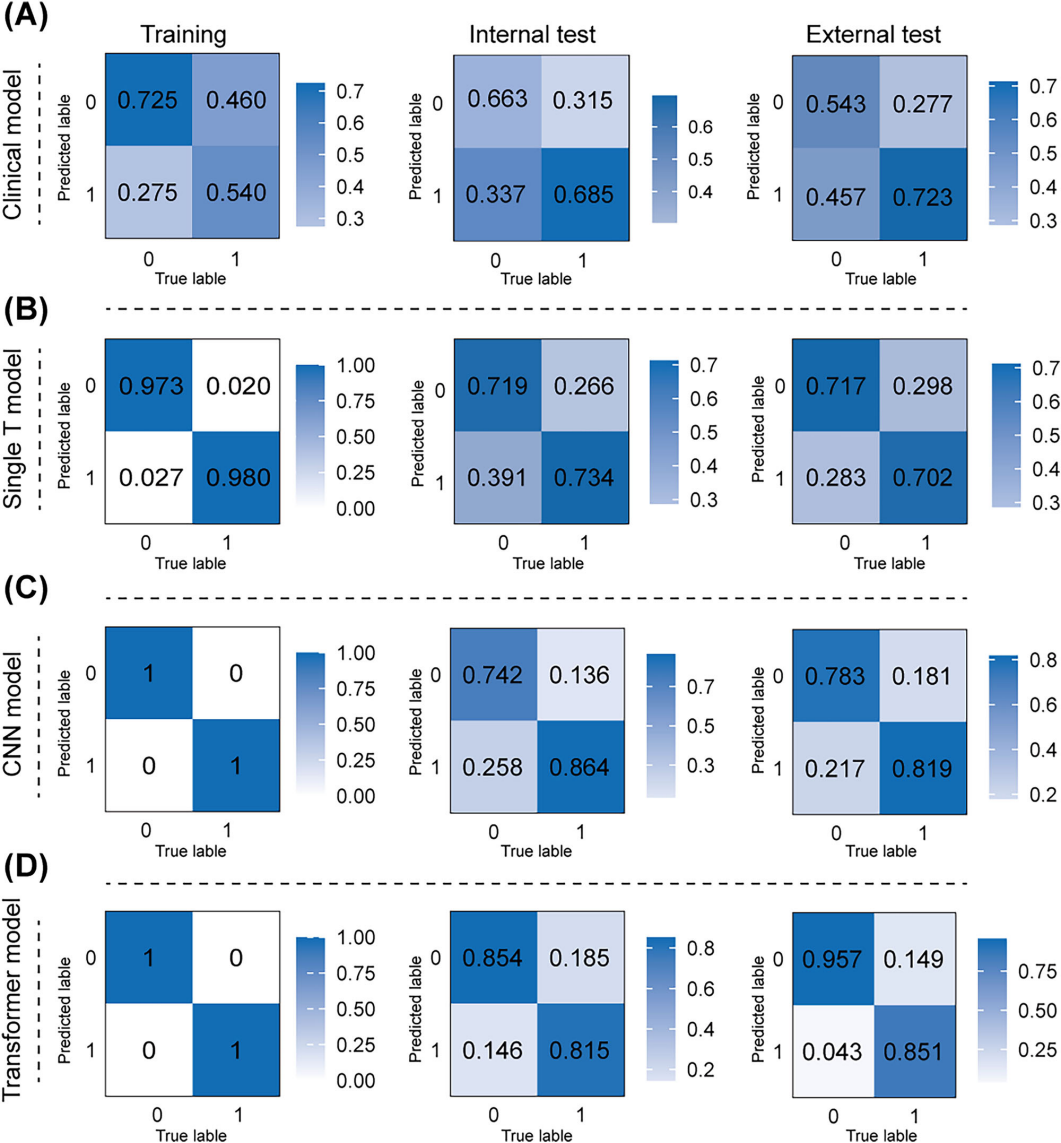
Confusion matrices of different DL models in all three cohorts. Confusion matrices of ALN status prediction of the clinical model (**A**), single T model (**B**) and DL framework employing CNN HRNet (**C**), and Transformer Segformer (**D**) as the network backbone in the training, internal test and external test cohorts. ALN axillary lymph node, DL deep learning, CNN convolutional neural network.

**Fig. 3:**
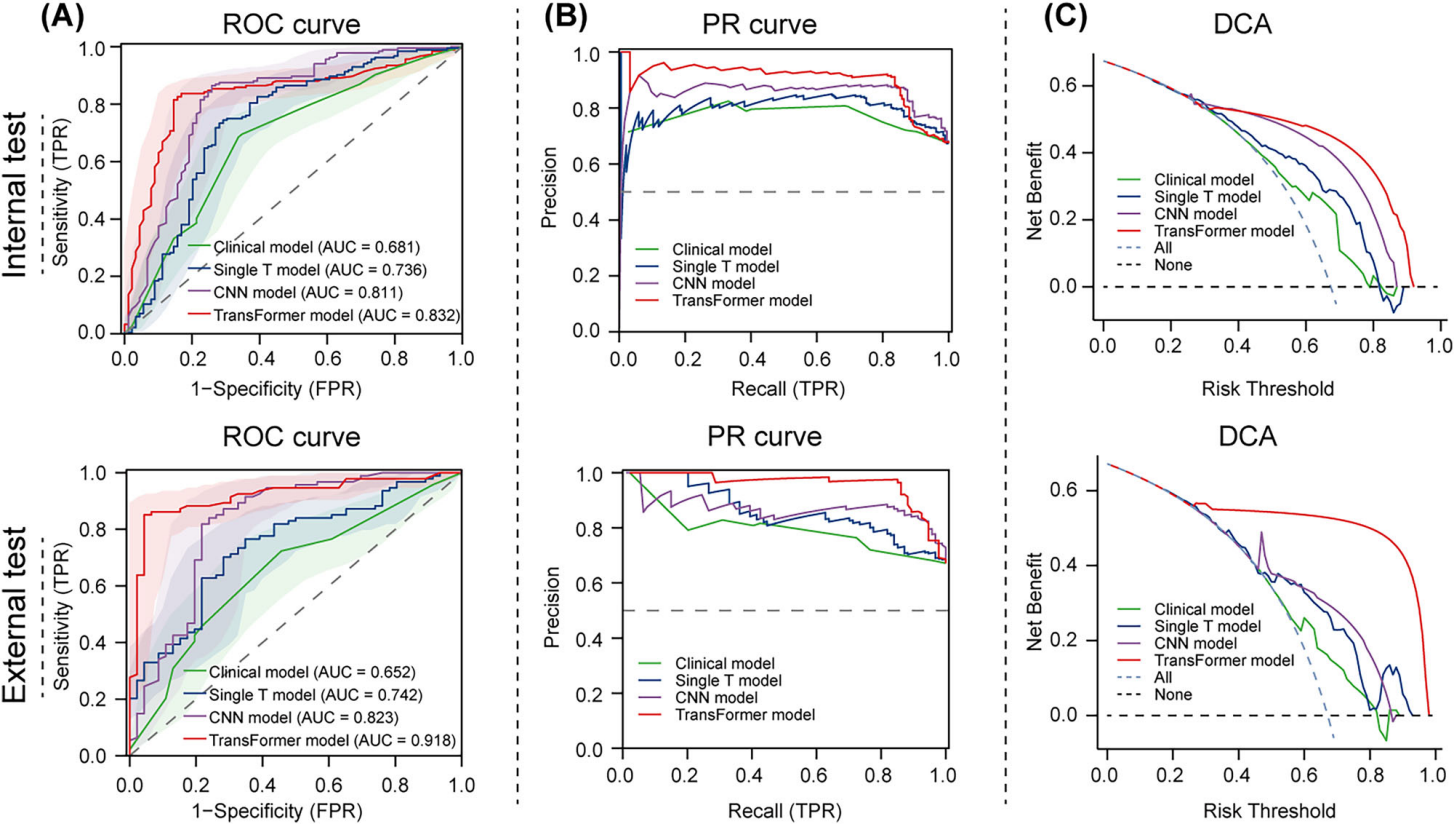
Confusion matrices of different DL models in all three cohorts. Evaluation of the performance of different DL models using ROC, PR and DCA analyses. ROC (**A**), PR (**B**) and DCA (**C**) curves comparing different models to diagnose ALN status in the internal and external test cohorts. ROC receiver operating characteristic, PR Precision-Recall, DCA decision curve analysis.

The confusion matrices of the clinical model, the single T model, CNN model (HRNet), and Transformer model (Segformer) in the training, internal, and external test cohorts were shown in Fig. 2. For the internal test cohort, the mistake diagnostic rate was 0.337 (clinical model), 0.391 (single T model), 0.258 (CNN model) and 0.146 (Transformer model). The omission diagnostic rates were relatively higher for the clinical (0.315) and single T model (0.266) compared to the CNN (0.136) and Transformer model (0.158). In the external test cohort, the Transformer model exhibited an excellent discrimination ability, with a mistake diagnostic rate of 0.043 and an omission rate of 0.149. To further analyze these models, ROC, PR and DCA curves were plotted (Fig. 3). Both ROC and PR analyses suggested the superior performance of the Transformer model compared to the other two models (Fig. 3A, B). According to the results of the DCA, if the threshold probability was greater than 0.43 in the internal test cohort and 0.27 in the external cohort, using the Transformer model to predict ALN metastasis gains more benefits than treat-all or treat-none tactics and more benefits than the clinical and CNN models (Fig. 3C). Taken together, we propose that the MTL and MIL framework using Segformer, one of the Transformer models, as the backbone for image feature extraction exhibited the best performance and clinical benefit and can be potentially utilized for further clinical application.

### Activation heat maps from ultrasound images

**Fig. 4:**
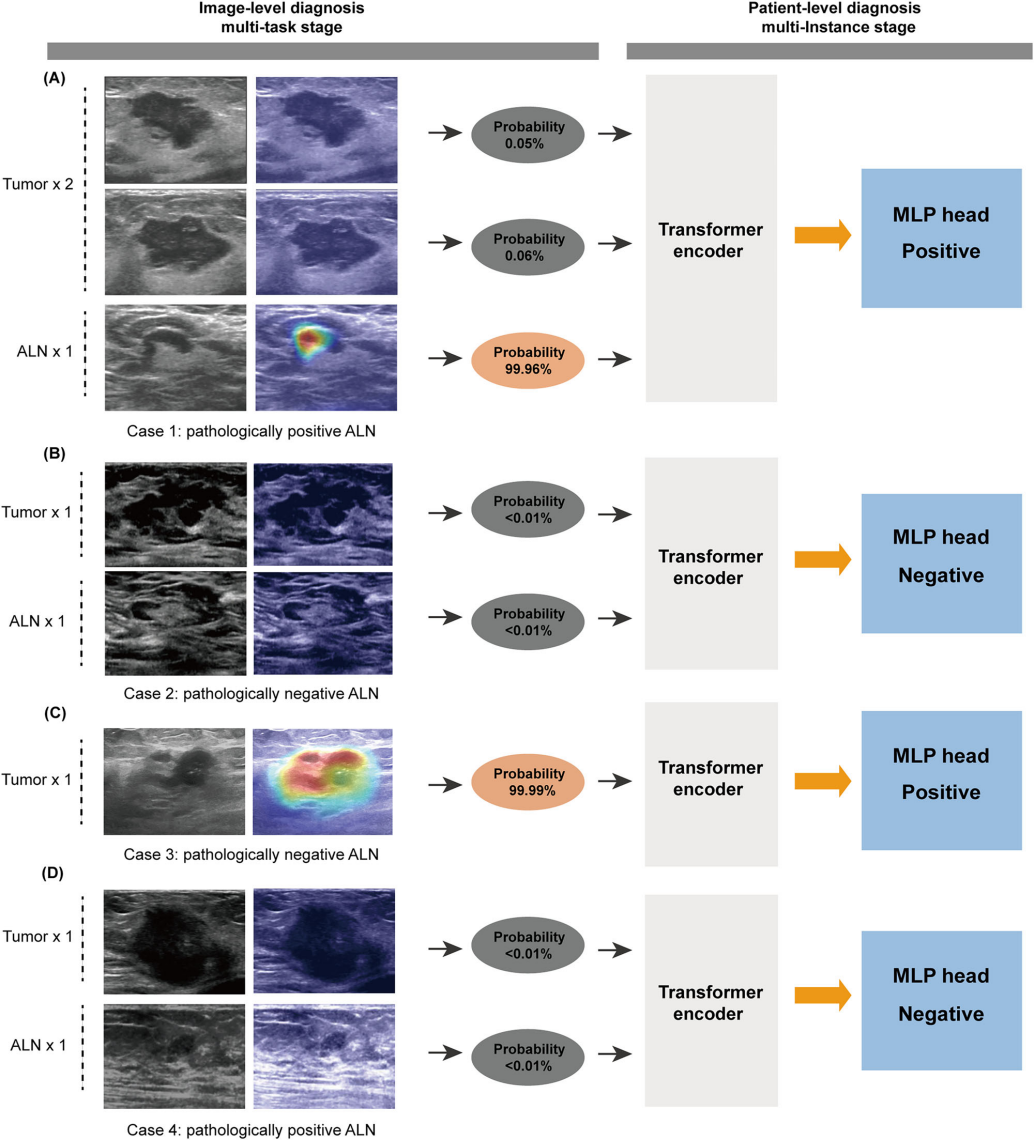
Case representation in the internal test cohort. The B-mode ultrasound images (primary tumor with or without ALNs and heat maps are displayed, with the diagnostic process shown. Examples of true positive (**A**), true negative (**B**), false positive (**C**), and false negative (**D**) cases were shown, respectively.

For better interpreting the strategy of this MTL and MIL framework in predicting LNM, and determining whether it focuses on the internal features of the tumor and ALN regions, we generated heat maps of both the primary and lymph node images from the Transformer (Segformer) model using the CAM method (Fig. 4). The CAM is applied to the classification head (CLS head), which consists of a GAP layer followed by a 1 × 1 convolutional layer. The activated regions labeled by different colors represent areas to which the model pays attention when making a prediction; in other word, they are areas that hold significance for ALN status prediction. Regions activated with red and yellow highlight characteristics highly suspicious for the ALN metastasis diagnosis, while the green and blue backgrounds reflect that the DL model didn’t recognize significant areas predictive for ALN metastasis. The deeper the color of a feature in an area, the more attention the DL model pays to it for prediction.

As shown in Fig. 4, four representative cases from the internal test set are exhibited. For case 1, two ultrasound images showing the tumor lesion from different angles and one image of the axillary area were utilized as input for the MTL stage. The heat map shows red and yellow activation areas in the ALN but not in the tumor lesion. Then the resulting image tokens generated by the MTL further served as input for the MIL and generated a final label of positive ALN, which is consistent with this patient’s pathological results (Fig. 4A). The case 2 had two ultrasound images respectively corresponding to the tumor and ALN area that could be inputted for the MTL; CAM analysis showed that no suspicious areas predictive for ALN metastasis were recognized by the DL model. Finally, the MIL generated a true label of negative ALN (Fig. 4B). We also showed examples of false positive (Fig. 4C) and false negative (Fig. 4D) cases, respectively. Besides, we compared the CAM patterns between the single-task classification model (the single T model) and our MTL model. As shown in Fig. S1, MTL effectively shifted the CAM focus from the tumor boundary and background to tumor center regions in these two cases.

## Discussion

In this study, we established an MTL and MIL DL framework that can simultaneously detect the primary tumor and ALN lesions on ultrasound images and predict ALN status, simulating the real-world clinical diagnostic scenarios. This design implicitly improves the model’s interpretability by constraining image features to lesion areas through the segmentation process and attention mechanisms. By introducing a segmentation task in the MTL stage and integrating tumor and ALN features from each patient, this model is guided to focus on areas within tumors and ALNs, preventing it from learning shortcuts and enabling a more comprehensive prediction using multi-view images. We demonstrated that the MTL and MIL DL frameworks, using different CNN and Transformer models as the network backbone, outperformed the clinical model and the single T model, highlighting the superiority of our DL model for predicting ALN metastasis. The DL framework using one of the Transformer models, Segformer, to extract image features, represents the top-performing model. It achieved an AUC of 0.832, a sensitivity of 0.815, and a specificity of 0.854 in the internal test cohort, and an AUC of 0.918, a sensitivity of 0.851, and a specificity of 0.957 in the external test cohort. The efficacy of this Transformer model is further supported by ROC, PR, and DCA analyses.

By offering direct visualization of the ALNs, ultrasound examination provides an effective way for physicians to diagnose ALN metastasis. However, naked-eye observation can sometimes overlook subtle ultrasound manifestations that hold clinical significance for discriminating metastasis. DL models, through training, can automatically extract image features and focus on lesion areas indicative of specific clinical outcomes through their attention mechanism, making them valuable second readers to provide additional opinions for physicians. However, the readability and interpretability of DL models pose significant challenges for clinical translation. It is often difficult to ascertain whether DL models have truly learned the essential disease-indicative regions or are predicting outcomes based on irrelevant features or shortcuts. In this study, we introduced instance segmentation—a computer vision task involving the identification and delineation of individual objects within an image^20,21^ —and attention mechanism into our model, which helps the classification head focus on lesion areas. This approach enables our model to simultaneously detect the regions of tumors and ALNs within images, and to predict patients’ ALN status. By employing this multi-task framework, the DL model is restricted to predicting ALN status based on the image features of the identified tumor and ALN regions. This prevents the model from learning shortcuts and might implicitly improve its interpretability, although it does not fundamentally resolve the black-box nature of deep learning models.

Previously, several studies reported on breast cancer DL models using ultrasound images to predict ALN statues. For instance, Zhou et al. reported a CNN model trained on primary breast cancer images from a cohort of 680 patients, achieving satisfactory AUCs of 0.805 and 0.720 in their internal and external test cohort, respectively^16^. However, their model is a single-task classification-only model that cannot detect tumor localization and region; this may add difficulties for interpretation and generalization. Based on smaller sample sizes, two other studies also constructed CNN models using suspicious lymph nodes seen on ultrasound or primary tumors^17,22^; however, their clinical translational potential is limited since their models were not tested on another cohort, and using suspicious lymph nodes alone for predicting patients’ ALN metastasis is not clinically practical because some patients, especially those with clinically negative breast cancer, might not have any visible lymph node detected by ultrasound examination. In our study, in addition to the MTL, we also introduced MIL, which integrated image features of both the primary tumor and ALN regions to make a final patient-level diagnosis. This DL model has a diagnostic strategy similar to that in the real-world clinical workflow, where clinicians first recognize the tumor and ALN lesions on an image, and then make a comprehensive judgment on patients’ ALN status based on their manifestations. This two-stage can be applied to a broader population, regardless of whether a patient has a visible ultrasound lymph node.

CNN and Transformer are commonly used network structures for image feature extraction in DL, each with its own advantages and disadvantages. CNN is an architecture based on convolutional layers, primarily used in the field of image processing for feature extraction. Over the last decade, CNNs have been a major focus of research in medical image analysis^23–25^. A majority of the published studies have adopted CNN structures for predicting lymph node metastasis and other clinical outcomes, such as biological characteristics, cancer histological subtypes, therapeutic outcome, and cancer prognosis, in breast cancer and other types of cancer^26–28^. However, the performance of CNNs may be limited by the inherent locality constraints and a resulting lack of explicit consideration of the long-range spatial relationships in an image^29,30^. Transformer is a DL structure based on the self-attention mechanism. Recently, Transformer architectures have been proposed to address the shortcomings of CNN and have gained increased attention in medical imaging tasks^31–33^. However, the application of Transformer models in medical binary tasks and their performance in comparison with CNN in predicting ALN metastasis in breast cancer patients remain largely unexplored. In this study, we also tested the predictive performance of distinct Transformer and CNN models as the network backbone in differentiating breast cancer patients with ALN metastasis. We showed that in the internal test cohort, the representative model of Transformer, Segformer, had a comparable AUC with the representative CNN model, HRNet (0.832 vs. 0.811; *p* = 0.565), but marginally outperform the HRNet model in the external test cohort (AUC, 0.918 vs. 0.823; *p* = 0.055). However, this subtle superiority might suffer from various real-world limitations, such as variances in image qualities and data distribution from the two different clinical centers.

Notably, the observed performance gap of the Segformer model on the external test set (AUC: 0.918) compared to the internal test set (AUC: 0.832) raises concerns about data distribution and potential overfitting. This discrepancy could stem from a range of real-world clinical factors of this retrospective study, such as variations in ultrasound equipment and acquisition protocols across different clinical centers, as well as differences in patient population—in this study, we found that the external test cohort included a higher proportion of hormone receptor-positive patients and more cases with lymphovascular invasion compared to the internal cohort (Table 1), both of which are known to associated with ALN status^34,35^. Moreover, this fact also indicates that our model might suffer from overfitting. This highlights the importance of further validation in future studies.

To understand the connections between DL model-extracted features and predictive outcomes, we adopted the CAM method to show the predictive parts with metastatic features on the image. The activation heat map shows our model focuses on features within the tumor and lymph node regions for ALN status prediction rather than irrelevant parts of the image. To some extent, the cases presented in Fig. 4 partially explain how the MTL and MIL methods are integrated to produce a final prediction. For example, our model correctly recognized case 1 as positive by integrating the image-level results, where the two primary breast cancer images were predicted to be negative, while the ALN was categorized as positive by the MTL. This indicates that our model might avoid false-negative results by combining predictive results of both the tumor and lymph node images. Indeed, statistical analysis revealed that the model using Segformer as the network backbone has a low false-negative rate, with 0.185 in the internal test cohort and 0.149 in the external cohort. We also show examples of false-negative and false-positive findings. The heat maps indicate that our model didn’t learn the features associated with ALN status in the two misdiagnosed cases. For example, while case 1 and case 4 both had ALN images for prediction, our model correctly recognized the positive ALN in case 1, but misdiagnosed the other case. This suggests that some image features with predictive ability haven’t been learned by our model, which also highlighted the importance of further prospective studies with larger sample sizes for advancing the DL model into clinical translation.

The ALN status is critical for breast cancer clinical management. ALN-positive patients often need more intensive therapeutic interventions, including neoadjuvant treatment, ALN dissection and postoperative radiotherapy. We here provide a DL model that helps predict ALN status before surgery, which can help clinician make therapeutic decisions. The top-performing DL model had a false positive rate of 0.146 and 0.043 for the internal and external cohort. This indicates that our model has a relatively low false-positivity that might cause overtreatment. Besides, our model might also be a potential tool for selecting patients for SLN (sentinel lymph node) biopsy. For patients with clinical negative ALN, SLN biopsy is recommended during surgery, which caused prolonged operative time and potential harm to patients. How to accurately identify patients who actually require SLN biopsy is an important clinical issue. Our model might be applicable in this setting: patients with clinically negativity but DL model-predictive positivity might be suitable for SLN biopsy. Moreover, our model also exhibits high specificity, with 0.854 and 0.957 in the internal and external test cohort, which means that it also performs well in preventing these negative patients from surgical dissection. Our model has false negative rates (0.185 in the internal cohort and 0.154 in the external cohort) that are in the range of 7.8–27.3%^36–38^, which is the reported false negative rates of the SLN dissection. Nonetheless, given the potential safety risks posed by false-negative results, clinical decision-making may require comprehensive consideration of additional patient characteristics—such as molecular subtype and T-stage—to ensure optimal outcomes.

Our study has several limitations. First, the retrospective nature of our study limited the robustness of our model for clinical application. The results reported in this study were dependent on the composition of the enrolled patient cohort, which may introduce sampling bias due to the relatively small and non-random sample. For translating our model into clinic, further prospective studies involving multiple centers and standardized imaging protocols are required, along with appropriate model calibration and improved DL model parameters tailored for realistic clinical practice. Secondly, although we meticulously compared the surgical pathological results with the ultrasound captured lymph nodes in patients enrolled in the training cohort and excluded lymph node images with uncertain pathological results, there is a low possibility of a negative lymph node being mistakenly labeled as positive and incorporated into the DL training process. This also highlights the necessity of conducting prospective studies in the future. Moreover, to improve the diagnostic efficiency and clinical application of DL models, more advanced DL method, such as CNN combining with Transformer, should be explored for predicting breast cancer ALN metastasis to determine the most suitable DL models for clinical application. Moreover, we used attention-based method in the MIL step considering the clinical application scenario; However, MIL has multiple variations, including instance-based, embedding-based, and attention-based method. Whether other models are more suitable and would improve our model’s performance needs further studies to elucidate.

In conclusion, we have demonstrated that using the MTL and MIL framework that simulates the real-world clinical diagnosis scenario can predict breast cancer ALN metastasis based on ultrasound images. This model exhibits favorable discrimination abilities and has a great potential facilitating the decision-making process of ALN management. Additionally, we compared CNN and Transformer models for image feature extraction. We found that the difference in their abilities to predict ALN metastasis is not particularly significant. However, despite this, Segformer demonstrates good performance in our study, highlighting its potential for translating into clinical application.

## Methods

### Patients and study design

Patients from two medical centers, the Second Xiangya Hospital (from January 1, 2019 and December 31, 2022) and Henan Cancer Hospital (from July 1, 2022 to December 31, 2022) were screened, and those who meet the eligibility criteria were enrolled for further investigation. This retrospective multi-cohort study received approval from the institutional review board of the Second Xiangya Hospital of Central South University (No. 2022064) and the institutional review board of the Affiliated Cancer Hospital of Zhengzhou University & Henan Cancer Hospital (No. 2017407). The study was performed in accordance with the Declaration of Helsinki. The requirement for informed consent was waived as we solely utilized anonymized retrospective data. The enrollment criteria were as follows: (1) patients pathologically diagnosed with unilateral breast cancer; (2) patients who underwent ultrasound examination of both the breast and axillary sites prior to biopsies/surgeries; (3) Patients diagnosed with early-stage breast cancer and eligible for surgery resection. Patients’ ALN status was confirmed by pathological examination on ALN tissues from biopsy or surgical resection, which is the clinical gold standard. The diagnosis is made by the Pathology departments of the respective hospitals. Patients with bilateral BC, distant metastases, or concurrent other types of cancer were excluded.

**Fig. 5:**
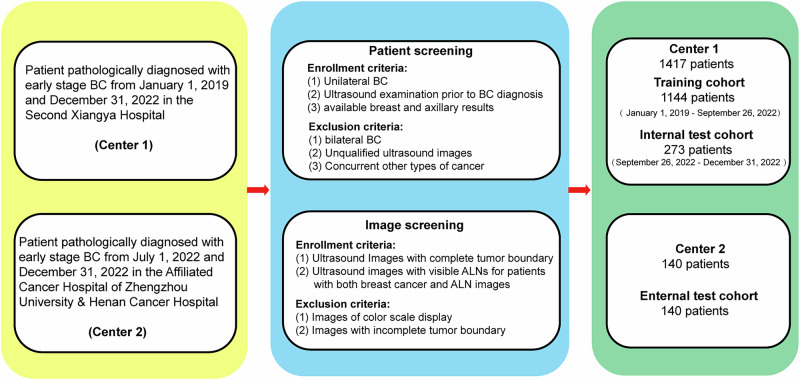
A flowchart illustrating the enrollment process of this study. BC breast cancer, ALN axillary lymph node.

Patients were stratified into three cohorts: a training cohort, an internal test cohort, and an external test cohort. The training cohort (*n* = 1144) and the internal test cohort (*n* = 273) comprised patients from the Second Xiangya Hospital, while patients (*n* = 140) from Henan Cancer Hospital constituted the external test cohort. In real-world scenarios, new patients are admitted sequentially over time. Therefore, to ensure relative independence of different cohorts, we divided patients into training and internal test cohorts based on their admission time with a ratio of 8:2. We sorted patients based on the initial admission time and checked their admission numbers to ensure that there were no duplicate patients. A flowchart illustrating the enrollment process is presented in Fig. 5.

Ultrasound images of both the breast and axillary sites were screened for each patient. For patients with unilateral multifocal BC, images of each tumor lesion were collected. For patients with unifocal BC, images from different perspectives were selected. For patients who had no visible ALNs on ultrasound images, only the primary breast cancer images were collected. In total, the primary tumor and ALN image numbers were 1875 and 970, 405 and 183, 221 and 126, in the training, internal test and external test cohorts, respectively. Breast cancer and ALN lesions were manually delineated using a Python-based graphical image annotation tool called LabelMe; these delineations were used as labels for training and evaluating the performance of the instance segmentation task. In the Second Xiangya Hospital, ultrasound equipment manufactured by Philips (Amsterdam, the Netherlands; EPIQ7), Siemens (Munich, Germany; S3000), Mindray (Shenzhen, China; R9), and GE Healthcare (Pittsburgh, PA; LOGIQ E9) was used to generate the Ultrasound images. In the Henan Cancer Hospital, ultrasound equipment manufactured by Philips (Amsterdam, the Netherlands; IU Elite, EPIQ5 and EPIQ7), and Mindray (Shenzhen, China; R9) was used to generate the ultrasound images. In both centers, the breast cancer two-dimensional mode was used for detection.

### Construction of the two-stage DL framework

**Fig. 6:**
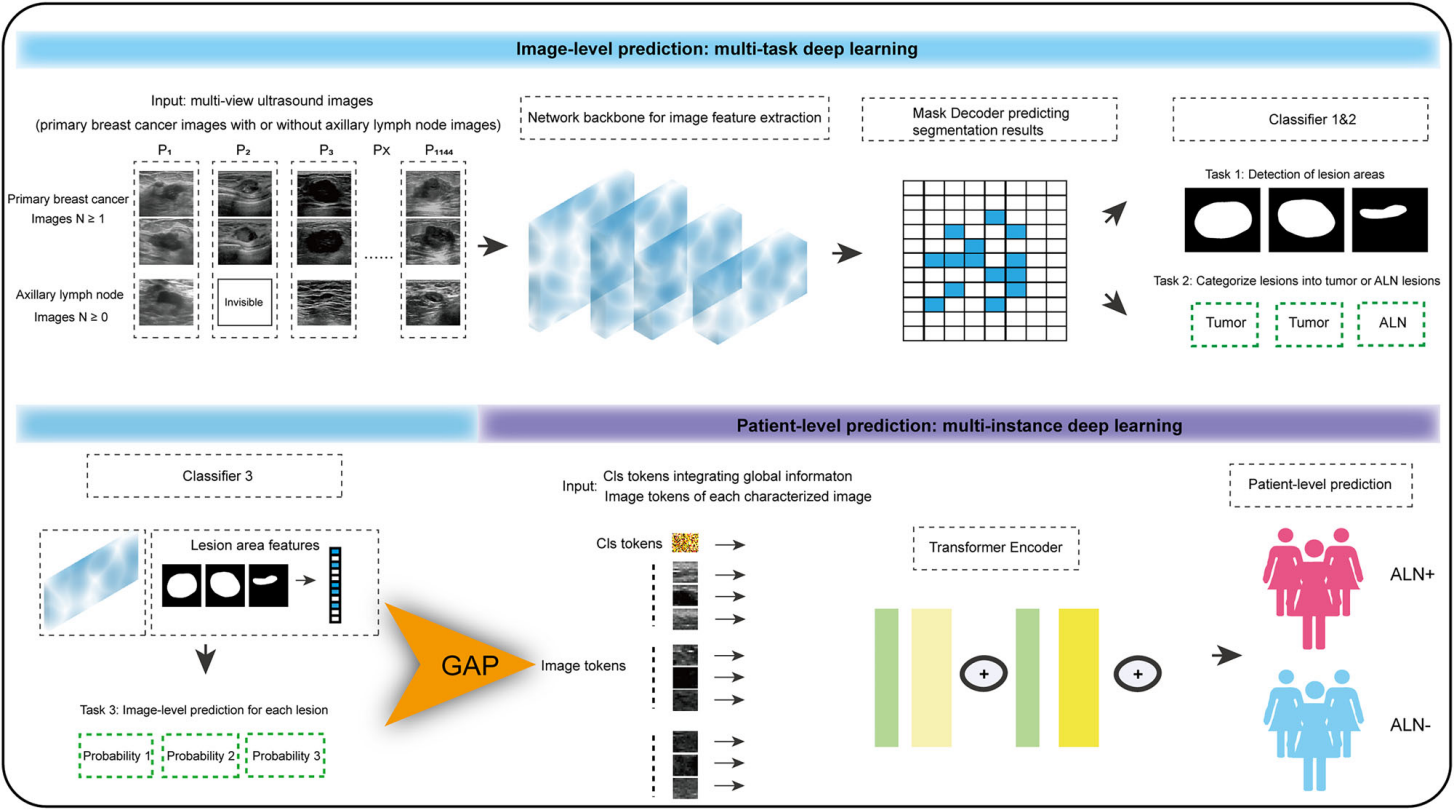
Overall two-stage deep learning system for breast cancer ALN status prediction. The first stage is an image-level multi-task learning stage. For each patient, primary tumor with or without ALN images was used as input for image feature extraction and segmentation. Three classifiers were applied to complete the following tasks: segmenting each lesion, categorizing each lesion as primary breast cancer or lymph node, and predicting lesion status on each image. The second stage is a patient-level multi-instance learning stage: a Transformer-based approach leverages multi-view features from the multiple images of one patient to obtain a comprehensive classification result. ALN axillary lymph node.

In this work, we propose a two-stage DL framework for predicting ALN status in breast cancer patients (Fig. 6):

(1) Image-level MTL stage: multiple images first went through feature extraction and mask decoder, then three classifiers were applied to complete the following tasks: segmenting each lesion, categorizing each lesion as primary breast cancer or lymph node, and predicting ALN status based on each image. To constrain the model’s learning to clinically significant regions, we integrated lesion segmentation into the prediction process. By applying masking and feature extraction based on the segmentation output, the model’s input was effectively constrained to the detected lesion regions. This design helps reduce the likelihood of learning shortcuts that might occur in classification-only models.

(2) Patient-level MIL stage: we adopted a Transformer-based approach to replace the simple rule-based method for predicting patients’ ALN status. Each patient is represented as a bag consisting of multiple images. These images originate from the same patient and share inherent interrelations. Therefore, it often requires integrating information across multiple instances to make a comprehensive diagnosis. Based on these considerations, we chose attention-based methods for MIL in our study. This approach leverages multi-view features from the images of one patient to complement lesion information, obtaining a more comprehensive classification result.

The detailed framework was shown in Fig. S2. In the image-level MTL stage, given a batch of input images including primary breast cancer and lymph node images, image feature *z* were extracted using the network backbone. Then, image feature passed through a mask decoder to further improve lesion extraction. Here, our mask decoder was implemented based on the SAM mask decoder. It took both the image features *z* and a learnable mask token as inputs and employed two-way attention to efficiently facilitate the interaction between the image feature and the mask token. The interacted image feature and the mask token went through transposed convolution and cross-attention respectively, to obtain the segmentation feature *s* and the enhanced mask token. Subsequently, these two features were fed into the segmentation head. The segmentation feature *s* underwent a dot product operation with the mask token that had been mapped through a MLP layer. Finally, a Sigmoid activation was adopted to obtain the segmentation result ${\hat{y}}_{{Seg}}$. With the segmentation result ${\hat{y}}_{{Seg}}$ and its corresponding Ground Truth (GT) ${y}_{{Seg}},$ Binary Cross-Entropy (BCE) loss was utilized to compute segmentation loss. Moreover, in order to help the model better concentrate on the lesion area, we used the segmentation result ${\hat{y}}_{{Seg}}$ as the attention map. This attention map describes the activation of lesion and non-lesion areas for ALN prediction and was subsequently multiplied with the segmentation feature *s* to yield mask feature ${z}_{m}$, a more complex feature focused on the lesion areas.

Then, a mask-classification head composed of a Global Average Pooling (GAP) layer and a 1 × 1 convolution layer was adopted to predict lesion classification result ${\hat{y}}_{{MCls}}$ (tumor or lymph node lesions). BCE loss was used to calculate the mask classification loss between ${\hat{y}}_{{MCls}}$ and the mask classification GT ${y}_{{MCls}}.$ Meanwhile, the segmentation result was further multiplied with the original image feature *z* to yield the lesion feature ${z}_{c}$. This feature could also help model better focus on lesion areas and thereby improve the accuracy of disease diagnosis. Then, a classification head composed of a GAP layer and a 1 × 1 convolution layer was used to predict ALN metastases ${\hat{y}}_{{Cls}}$ at an image-level (positive or negative). Similarly, the image-level classification loss was also calculated using the BCE loss. Here, the image-level labels ${y}_{{Cls}}$ were pseudo-labels generated based on the patient-level labels. These above losses jointly optimized the mask decoder and the backbone of the model. With enforced constraints via attention maps, the model can effectively focus on the lesion areas of each image, thus alleviating the issues learning shortcuts to some extent.

In the patient-level MIL stage, we first extracted the patient-level feature using the pretrained model. Given a bag of instances composed of M breast cancer images and N lymph node images for each patient, we adopted a GAP layer on the lesion feature ${z}_{c},$ which was multiplied by the original image feature *z* and the attention map, to extract image tokens. We organized these extracted image tokens into bags and input them into the Transformer-based multi-instance learning model. In the Transformer encoder, the CLS token was generated by random initialization, which was used to integrate global image information. The image tokens and the CLS token then underwent self-attention operations within the transformer encoder, where the CLS token attended to all other image tokens and integrate their information into itself. Finally, the CLS token passed through a straightforward MLP layer to predict patient-level ALN status. BCE loss was computed to optimize the multi-instance classification model. This attention-based multi-instance framework can consider the correlations between multi-view images, thus enabling a more comprehensive prediction.

Besides, for comparison, we followed the study by Zhou et al. and trained a Restnet101 model (the single T model) on our dataset^16^. This is a single-task classification model that used primary tumor images for ALN prediction.

### Implementation details

In the multi-task framework, we employ various CNN (HRNet^39^, ResNet^40^, Unet^41^ and MobileNet^42^) and Transformer (Swin^43^ and Segformer^44^) models as the backbone, with the mask decoder sourced from the SAM^45^ as the decoder. These CNN and Transformer models were tested individually and the network backbone only consisted of one of the models. Our mask decoder is similar to the implementation of SAM mask decoder. We discarded the IoU tokens and the corresponding MLP layer as we did not predict IoU scores. Then, we divided the mask decoder of SAM into two parts. The part without the mask token MLP and dot product at the front was used as our mask decoder to extract segmentation feature *s*, while the mask token MLP and dot product in the second half served as the segmentation head to predict the segmentation results. The segmentation feature *s* refers to the feature in the mask decoder that has gone through 2x transposed convolution layer.

Preprocessing steps involved firstly resizing images to a uniform resolution of 512 × 512 pixels with a ratio range of 0.8–1.25, followed by random cropping (a fixed-size 512 × 512 region) and flipping with a 50% probability. We adopted an online data augmentation randomly. The bag size was set to 16, with a total iteration of 10k. We utilized the AdamW optimizer with a learning rate of 6e-5 for model optimization. In the MIL stage, the token dimension was set to 256, and the Transformer is a basic ViT-base model^46^ with a simple MLP layer serving as the classifier. The bag size was set to 4, and the model was trained for a total of 100 epochs. In our model, all available image features from each patient are fed into the Transformer to enable inter-instance interaction without input length normalization, including cases where ALN images are missing. We generated heat maps of both the primary and lymph node images from the Transformer (Segformer) model using the class activation mapping (CAM) method. The CAM is applied to the classification head (CLS head), which consists of a GAP layer followed by a 1 × 1 convolutional layer. For the segmentation task, we evaluated performance using the commonly used mIoU metric (Table S2). The versions of Python and all major libraries are as follows: Python: 3.8.5; TorchVision: 0.15.2+cu117; OpenCV: 4.9.0; MMCV: 1.7.1; MMSegmentation: 0.16.0+; MMClassification: 0.18.0+.

### Clinical model construction

A clinical model for predicting breast cancer ALN metastasis was developed using Logistic regression analysis. To identify suitable factors for model construction, both univariate and multivariate logistic regression analyses were conducted on the training cohort, with the results presented in Table S3. Characteristics such as BI-RADS category and clinical T stage, which are obtainable before surgery and have been identified as significant risk factors for ALN metastasis, were included in the final clinical model (Fig. S3). This established model underwent testing in both the internal and external cohorts. The R package rms was utilized for model construction using the training cohort and subsequent testing on the internal and external cohorts.

### Analyses of ROC, PR and DCA

DeLong test was used to compare AUCs of different model. We used ROC and PR analyses for evaluating the performance of various prediction models. While the ROC curve considers both positive and negative cases, the PR curve primarily focuses on positive cases. DCA serves as a tool for measuring the effectiveness of medical decisions by comparing threshold values with net benefits, thus allowing for an assessment of the merits of different decision strategies. In this study, we employed DCA to compare the clinical benefits of different models. The R package pROC was used for ROC and PR analysis; the R package rmda was used to calculate net benefit and calibration plot; The R package ggplot2 was used for visualization.

### Statistical analysis

The chi-square test was used to assess the association of clinical characteristics with ALN status in breast cancer patients. The DeLong test was employed to compare the area under the curve (AUC) of different prediction models. The significance level was set at 0.05 for all of the analyses. Statistical calculations in this study were performed using R software (R 4.0.3).

## Supplementary information

Supplementary Materials

checklist

## Data Availability

The original ultrasound images and clinical data used in this study are not publicly available due to the restrictions of hospital regulations and patient privacy. Other data supporting the main findings of this study are available on request for non-commercial purposes from the corresponding authors Y.C. and Y.X.L. typically within 2 weeks.

## Code availability

The code will be available at https://github.com/Ferraaaaa/BC_ALN_MTL_MIL.
