# Supplementary material for: Applying a multi-task and multi-instance framework to predict axillary lymph node metastases in breast cancer
Source: NPJ Precis Oncol. 2025 Jun 18;9:195. doi: 10.1038/s41698-025-00971-0 (PMC12177086; doi:10.1038/s41698-025-00971-0)
Supplement: Supplementary file 1 — Supplementary Materials [file 41698_2025_971_MOESM1_ESM.pdf]

Supplementary figures

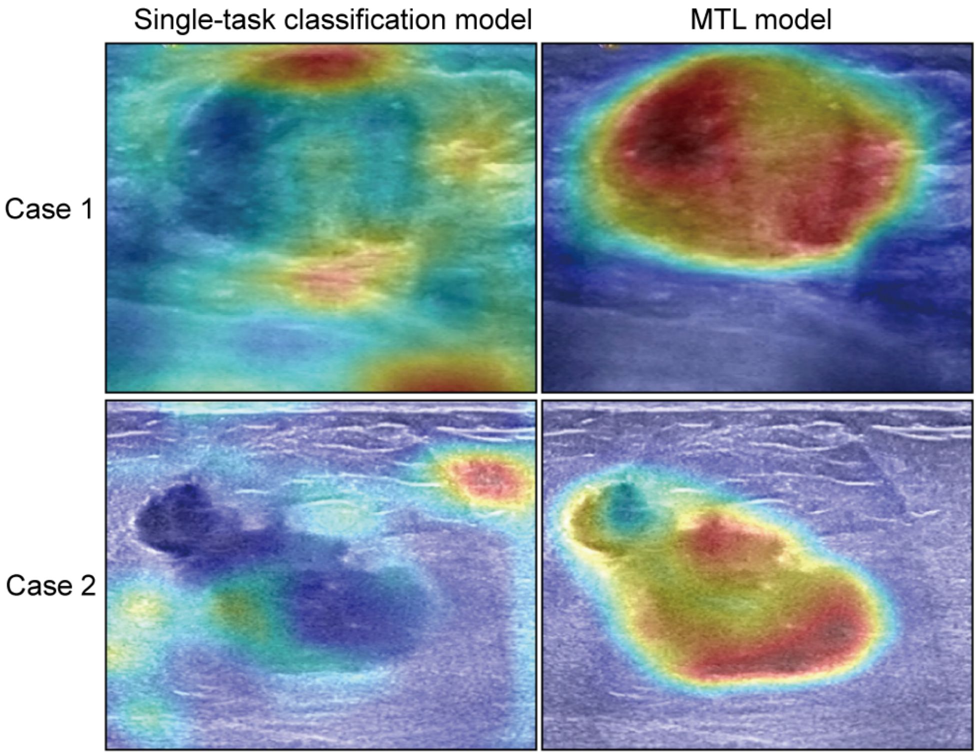

Figure S1. Comparison of the CAM patterns of the single-task classification model and the MTL model. Two representative cases are shown

Multi-Task Framework for Image-Level Diagnosis

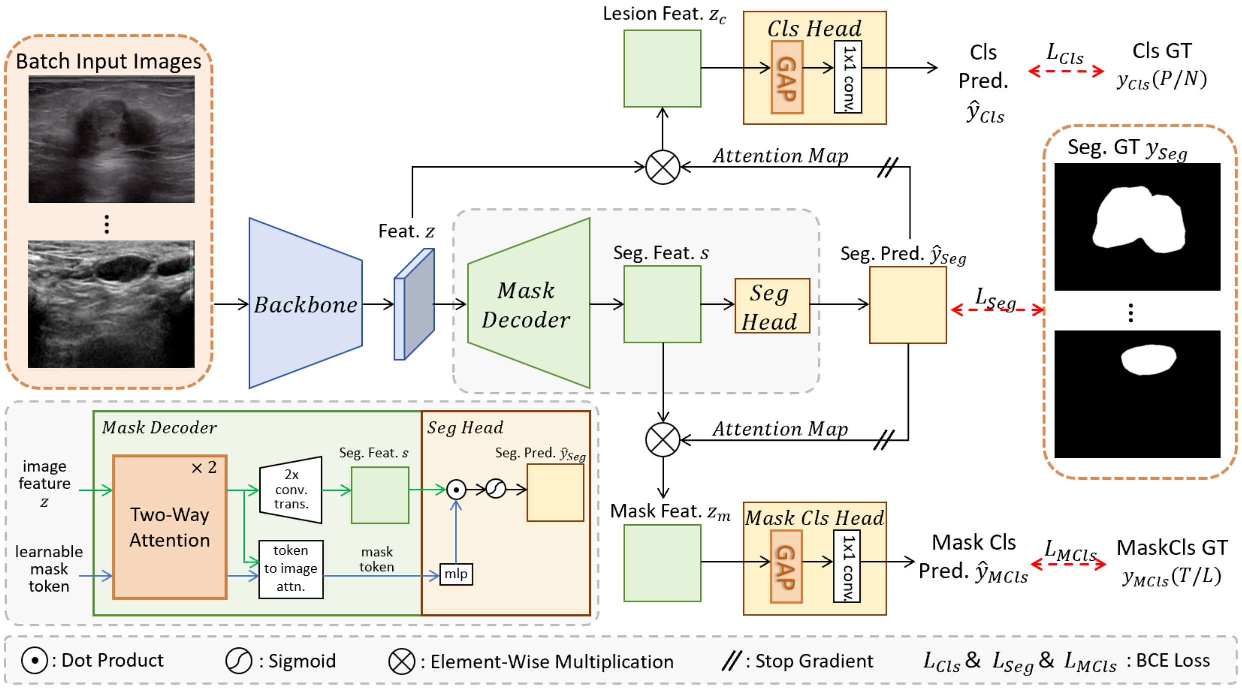

Multi-Instance Framework for Patient-Level Diagnosis

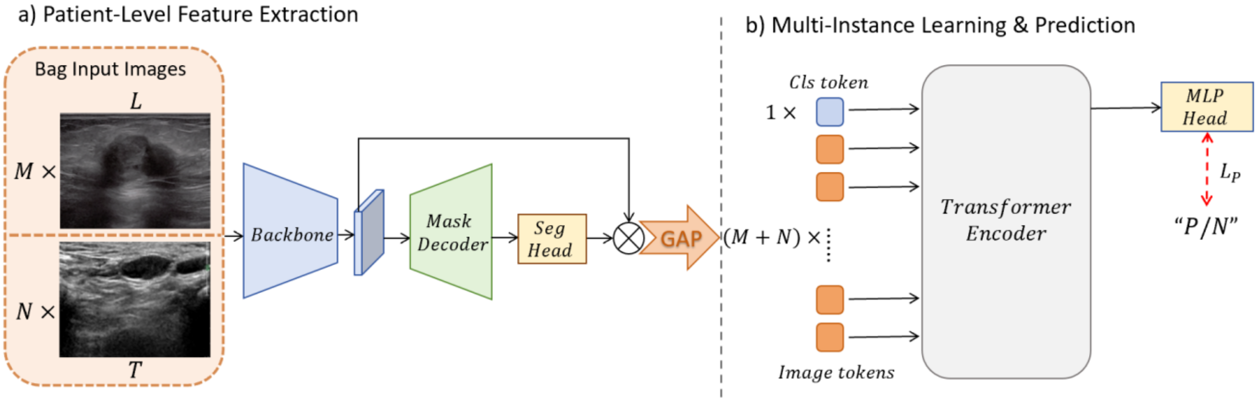

Figure S2. The two-step DL framework information**.** P/N, positive/negative; T/L, tumor/lymph node.

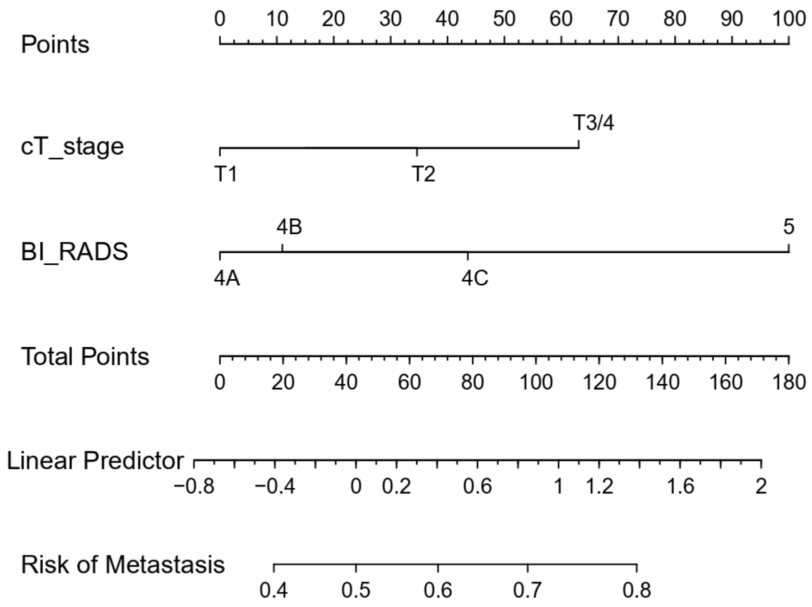

Figure S3. The clinical nomogram model was developed using preoperative indicators including clinical T stage and ultrasound reported BI-RADS category. BI-RADS, breast imaging reporting and data system.

Supplementary tables

Table S1. Distribution of clinical characteristics in patients with distinct axillary lymph node status

	Training cohort (N=1144)			Internal test cohort (N=273)			External test cohort (N=140)		
Characteristic	Positive, N (%)	Negative, N (%)	P value	Positive, N (%)	Negative, N (%)	P value	Positive, N (%)	Negative, N (%)	P value
No. of patients (%)	735 (64.2%)	409 (35.8%)		184 (67.4%)	89 (32.6%)		94 (67.1%)	46 (32.9%)	
Age, median (IQR) [a]	51 (44, 57)	50 (44, 57)	0.477	50.59 ± 10.01	48.90 ± 8.53	0.174	52.649 ± 9.0193	51.174 ± 10.777	0.396
Clinical T stage			**< 0.001**			**< 0.001**			0.245
T1	193 (26.3%)	156 (38.1%)		46 (25.0%)	46 (51.7%)		34 (36.2%)	21 (45.7%)	
T2	472 (64.2%)	225 (55.0%)		126 (68.5%)	38 (42.7%)		56 (59.6%)	25 (54.3%)	
T3	51 (6.9%)	15 (3.7%)		11 (6.0%)	4 (4.5%)		4 (4.3%)	0 (0.0%)	
T4	19 (2.6%)	13 (3.2%)		1 (0.5%)	1 (1.1%)		0 (0.0%)	0 (0.0%)	
US reported BI-RADS			**< 0.001**			**< 0.001**			0.056
4A	28 (3.7%)	38 (9.3%)		14 (7.6%)	15 (16.9%)		9 (9.6%)	10 (21.7%)	
4B	109 (14.8%)	118 (28.9%)		25 (13.6%)	26 (29.2%)		11 (11.7%)	5 (10.9%)	
4C	269 (36.6%)	169 (41.3%)		74 (40.2%)	29 (32.6%)		45 (47.9%)	25 (54.4%)	
5	329 (44.8%)	84 (20.5%)		71 (38.6%)	19 (21.3%)		29 (30.9%)	6 (13.0%)	
Pathological type			**< 0.001**			0.116			0.605
Invasive ductal	629 (85.6%)	295 (72.1%)		163 (88.6%)	70 (78.7%)		69 (73.4%)	33 (71.7%)	
Invasive lobular	13 (1.8%)	9 (0.8%)		2 (1.1%)	4 (4.5%)		4 (4.3%)	1 (2.2%)	
Invasive cancer mixed with in situ cancer	80 (10.9%)	91 (22.2%)		14 (7.6%)	11 (12.3%)		19 (20.2%)	12 (26.1%)	
Others	13 (1.8%)	14 (3.4%)		5 (2.7%)	4 (4.0%)		2 (2.1%)	0 (0.0%)	
Receptor status									
ER status			0.148			0.237			0.098
Positive	486 (66.1%)	253 (61.9%)		125 (67.9%)	54 (60.7%)		77 (81.9%)	32 (69.6%)	
Negative	249 (33.9%)	156 (38.1%)		59 (32.1%)	35 (39.3%)		17 (18.1%)	14 (30.4%)	
PR status			0.331			0.204			0.068
Positive	394 (53.6%)	207 (50.6%)		108 (58.7%)	45 (50.6%)		73 (77.7%)	29 (63.0%)	
Negative	341 (46.4%)	202 (49.4%)		76 (41.3%)	44 (49.4%)		21 (22.3%)	17 (37.0%)	
CerbB-2 status			0.314			0.159			0.258
Positive	252 (34.3%)	153 (37.4%)		52 (28.3%)	33 (37.1%)		40 (42.5%)	15 (32.6%)	

Negative	483 (65.7%)	256 (62.6%)		132 (71.7%)	56 (62.9%)		54 (57.5%)	31 (67.4%)	
Ki67 expression			0.267			**0.006**			0.911
1-10%	58 (7.9%)	44 (10.8%)		16 (8.7%)	18 (20.2%)		3 (3.2%)	2 (4.3%)	
11-50%	501 (68.2%)	275 (67.2%)		120 (65.2%)	58 (61.2%)		60 (63.8%)	30 (65.2%)	
51-100%	176 (23.9%)	90 (22.0%)		48 (26.1%)	13 (14.6%)		31 (33.0%)	14 (30.4%)	
Lymphovascular invasion [b]			**< 0.001**			**< 0.001**			**< 0.001**
Yes	206 (54.8%)	73 (27.0%)		60 (61.9%)	5 (8.9%)		64 (81.0%)	8 (21.0%)	
No	170 (45.2%)	197 (73.0%)		37 (38.1%)	51 (91.1%)		15 (19.0%)	31 (79.0%)	
Perineural invasion [b]			0.223			**0.030**			0.561
Yes	53 (7.2%)	32 (7.8%)		16 (17.8%)	3 (5.4%)		7 (7.4%)	2 (4.3%)	
No	297 (40.4%)	240 (58.7%)		74 (82.2%)	53 (94.6%)		45 (47.9%)	28 (60.9%)	

[a] T test; [b] Incomplete data. IQR, interquartile range; BI-RADS, breast imaging reporting and data system.

Table S2. Segmentation results of the model

Experimental design<br>10k，bs=16,512x512				
Methods	Segmentation (Internal test cohort)		Segmentation (External test cohort)	
	Tumor lesion IoU	ALN lesion IoU	Tumor lesion IoU	ALN lesion IoU
HRNet	71.86	63.20	73.90	56.80
ResNet	70.43	60.07	75.27	54.48
Unet	60.63	41.23	62.23	33.39
MobileNet	66.64	50.56	68.76	32.15
Swin	72.21	59.85	73.00	50.09
Segformer	72.61	63.53	75.94	48.52

ALN, axillary lymph node; IoU, intersection of union.

Table S3. Logistic analysis of axillary lymph node metastasis risk and clinical-pathological characteristics

		Univariate analysis		Multivariate analysis	
	Total (N)	OR (95% CI)	P value	OR (95% CI)	P value
		**Before surgery**			
Age, years	1144	1.005 (0.993 - 1.018)	0.403		
BI-RADS category	1144				
4A	66	Reference		Reference	
4B	227	1.254 (0.721 - 2.180)	0.423	0.800 (0.386 – 1.661)	0.550
4C	438	2.160 (1.278 - 3.651)	**0.004**	1.134 (0.567 – 2.266)	0.722
5	413	5.315 (3.086 - 9.156)	**< 0.001**	2.188 (1.056 – 4.535)	**0.035**
Clinical T stage	1144				
T1	349	Reference		Reference	
T2	697	1.696 (1.302 - 2.208)	**< 0.001**	1.189 (0.813 – 1.738)	0.373
T3	66	2.748 (1.489 - 5.074)	**0.001**	2.659 (1.055 – 6.703)	**0.038**
T4	32	1.181 (0.566 - 2.467)	0.657	0.948 (0.341 – 2.638)	0.919
		**After surgery**			
Pathological type	1144				
Invasive ductal	924	Reference		Reference	
Invasive lobular	22	0.677 (0.286 - 1.603)	0.375	0.920 (0.301 – 2.810)	0.884
Invasive mixed with in situ	171	0.412 (0.296 - 0.574)	**< 0.001**	0.484 (0.303 – 0.774)	**0.002**
Others	27	0.435 (0.202 - 0.938)	**0.034**	0.389 (0.102 – 1.493)	0.169
Vascular invasion	646				
Negative	367	Reference		Reference	
Positive	279	3.270 (2.335 - 4.579)	**< 0.001**	3.046 (2.145 – 4.327)	**< 0.001**
Nerve invasion	622				
Negative	537	Reference			
Positive	85	1.338 (0.836 - 2.143)	0.225		
ER status	1144				
Negative	405	Reference			
Positive	739	1.203 (0.936 - 1.547)	0.149		
PR status	1144				
Negative	543	Reference			
Positive	601	1.128 (0.885 - 1.436)	0.331		
CerbB-2	1144				
Negative	739	Reference			
Positive	405	0.873 (0.679 – 1.123)	0.290		
Ki67	1144				
1-10%	102	Reference		Reference	
11-50%	776	1.382 (0.909 - 2.100)	0.130	0.664 (0.348 – 1.266)	0.214
51-100%	266	1.484 (0.930 - 2.366)	**0.098**	0.577 (0.285 – 1.169)	0.127

OR, odd ratio; CI, confidence interval; BI-RADS, breast imaging reporting and data system.
