# Supplementary material for: Applying a multi-task and multi-instance framework to predict axillary lymph node metastases in breast cancer
Source: NPJ Precis Oncol. 2025 Jun 18;9:195. doi: 10.1038/s41698-025-00971-0 (PMC12177086; doi:10.1038/s41698-025-00971-0)
Supplement: Supplementary file 2 — checklist [file 41698_2025_971_MOESM2_ESM.pdf]

Checklist for Artificial Intelligence in Medical Imaging (CLAIM): 2024 Update

Section / Topic	No.	Item	Page / Line	No	NA
**TITLE / ABSTRACT**					
	**1**	Identification as a study of AI methodology, specifying the category of technology used (e.g., deep learning)	1		
**ABSTRACT**					
	**2**	Summary of study design, methods, results, and conclusions	27-38		
**INTRODUCTION**					
	**3**	Scientific and/or clinical background, including the intended use and role of the AI approach	41-89		
	**4**	Study aims, objectives, and hypotheses	90-102		
**METHODS**					
Study Design	**5**	Prospective or retrospective study	344-345		
	**6**	Study goal	381-382		
Data	**7**	Data sources	342-344		
	**8**	Inclusion and exclusion criteria	349-356		
	**9**	Data pre-processing	454-456		
	**10**	Selection of data subsets	360-364		
	**11**	De-identification methods	398-438		
	**12**	How missing data were handled	460-462		
	**13**	Image acquisition protocol	373-379		
Reference Standard	**14**	Definition of method (s) used to obtain reference standard	352-353		
	**15**	Rationale for choosing the reference standard	354		
	**16**	Source of reference standard annotations	354-355		
	**17**	Annotation of test set	354-355		
	**18**	Measures of inter- and intra-rater variability of features described by the annotators			NA
Data Partitions	**19**	How data were assigned to partitions	360-362		
	**20**	Level at which partitions are disjoint	362-364		
Testing Data	**21**	Intended sample size	358-359		

Section / Topic	No.	Item	Page / Line	No	NA
Model	**22**	Detailed description of model	398-438		
	**23**	Software libraries, frameworks, and packages	466-468		
	**24**	Initialization of model parameters	432		
Training	**25**	Details of training approach	415-425		
	**26**	Method of selecting the final model	489-490		
	**27**	Ensembling techniques	398-438		
Evaluation	**28**	Metrics of model performance	478-485		
	**29**	Statistical measures of significance and uncertainty	489-490		
	**30**	Robustness or sensitivity analysis			NA
	**31**	Methods for explainability or interpretability	386-390		
	**32**	Evaluation on internal data	478-486		
	**33**	Testing on external data	478-486		
	**34**	Clinical trial registration			NA
**RESULTS**					
Data	**35**	Numbers of patients or examinations included and excluded	105		
	**36**	Demographic and clinical characteristics of cases in each partition	105-113		
Model performance	**37**	Performance metrics and measures of statistical uncertainty	124-157		
	**38**	Estimates of diagnostic performance and their precision	158-175		
	**39**	Failure analysis of incorrect results			NA
**DISCUSSION**					
	**40**	Study limitations	315-332		
	**41**	Implications for practice, including intended use and/or clinical role	297-314		
**OTHER INFORMATION**					
	**42**	Provide a reference to the full study protocol or to additional technical details	504-505		
	**43**	Statement about the availability of software, trained model, and/or data	500-503		
	**44**	Sources of funding and other support; role of funders	494-498		

* Indicate page and/or line number for each checklist item that is present. NA = not applicable.

Tejani AS, Klontzas ME, Gatti AA, et al. Checklist for Artificial Intelligence in Medical Imaging (CLAIM):2024 Update. Radiol Artif Intell 2024;6(4):e240300. https://doi.org/10.1148/ryai.240300
